# Supplementary material for: AAV-mediated base-editing therapy ameliorates the disease phenotypes in a mouse model of retinitis pigmentosa
Source: Nat Commun. 2023 Aug 15;14:4923. doi: 10.1038/s41467-023-40655-6 (PMC10427680; doi:10.1038/s41467-023-40655-6)
Supplement: Supplementary file 1 — Supplementary Information [file 41467_2023_40655_MOESM1_ESM.docx]

**Supplementary Information**

**AAV-mediated base-editing therapy ameliorates the disease phenotypes in a mouse model of retinitis pigmentosa**

Yidong Wu^1,2,3#^, Xiaoling Wan^1,2,3#,*^, Dongdong Zhao^4,5#^, Xuxu Chen^4,5#^, Yujie Wang^4,5#^, Xinxin Tang^4,5^, Ju Li^6^, Siwei Li^4,5^, Xiaodong Sun^1,2,3*^, Changhao Bi^4,5*^ and Xueli Zhang^4,5*^

^1^Department of Ophthalmology, Shanghai General Hospital, Shanghai Jiao Tong University School of Medicine, Shanghai, China

^2^National Clinical Research Center for Eye Diseases, Shanghai, China

^3^Shanghai Key Laboratory of Ocular Fundus Diseases, Shanghai, China

^4^Tianjin Institute of Industrial Biotechnology, Chinese Academy of Sciences, Tianjin, China;

^5^National Technology Innovation Center of Synthetic Biology, Tianjin, China;

^6^College of Life Science, Tianjin Normal University, Tianjin, China;

^#^These authors contributed equally to this work

*Corresponding authors

Email: shaolin.72@163.com; xdsun@sjtu.edu.cn; bi_ch@tib.cas.cn; zhang_xl@tib.cas.cn

**Table of Contents**

[Supplementary Table 1. Frequency of pde6b allelic variants in vitro Cells with different sgRNAs (%) 3](#_Toc139558471)

[Supplementary Table 2. Frequency of pde6b allelic variants in the neuroretina of *rd10* mice (%) 4](#_Toc139558472)

[Supplementary Table 3. Editing efficiencies with A-to-G conversion at off-target loci in the neuroretina of rd10 mice (%) 5](#_Toc139558473)

[Supplementary Table 4. The main primers used for deep sequence 6](#_Toc139558474)

[Supplementary Table 5. The on-target and off-target sequence of Pde6b in *rd10* mouse 7](#_Toc139558475)

[Fig. S1 Preservation of cones in rd10 mice after ABE treatment at P35. 8](#_Toc139558476)

[Fig. S2 Rescue of retinal structure and visual function in rd10 mice after ABE treatment at P90. 9](#_Toc139558477)

[Fig. S3 Improvement of vision-guided behavior of rd10 mice after dual-AAV SpRY-ABE8e treatment at P90. 10](#_Toc139558478)

[Fig. S4 Quantification of scotopic b-wave amplitudes of A7-treated rd10 mice at different time points. 11](#_Toc139558479)

[Fig. S5 Histology of representative rd10 mouse retina from P1W to P4W. 12](#_Toc139558480)

# Supplementary Table 1. Frequency of pde6b allelic variants in vitro Cells with different sgRNAs (%)

|  | Unedited | Target Only | Target with Bystander | Bystander Only | Indels |
| --- | --- | --- | --- | --- | --- |
| sgRNA1 | 54.53±2.15 | 12.55±1.12 | 31.35±1.90 | 1.09±0.03 | 0.49±0.02 |
| sgRNA2 | 42.09±0.96 | 2.81±0.27 | 53.26±1.04 | 0.48±0.04 | 1.36±0.10 |
| sgRNA3 | 66.62±2.81 | 16.23±1.38 | 16.31±1.50 | 0.63±0.06 | 0.22±0.01 |
| sgRNA4 | 39.26±1.05 | 36.39±1.15 | 23.31±1.12 | 0.49±0.03 | 0.55±0.05 |
| sgRNA5 | 42.95±0.77 | 39.93±0.60 | 15.70±1.33 | 0.47±0.01 | 0.95±0.03 |
| sgRNA6 | 60.02±2.87 | 6.15±0.29 | 32.72±2.62 | 0.75±0.07 | 0.36±0.03 |
| sgRNA7 | 54.91±2.12 | 40.71±1.86 | 2.96±0.03 | 0.88±0.26 | 0.55±0.29 |

# Supplementary Table 2. Frequency of pde6b allelic variants in the neuroretina of *rd10* mice (%)

|  |  | Unedited | Target Only | Target with Bystander | Bystander Only | Indels |
| --- | --- | --- | --- | --- | --- | --- |
| Genome DNA | BSS | 98.86±0.06 | 0.30±0.01 | 0.01±0.01 | 0.55±0.03 | 0.29±0.04 |
|  | NT Treated | 98.60±0.11 | 0.37±0.03 | 0.04±0.03 | 0.65±0.07 | 0.34±0.04 |
|  | A7 Treated | 91.96±2.95 | 13.06±2.22 | 3.05±0.91 | 1.42±0.11 | 0.51±0.18 |
| cDNA | BSS | 98.92±0.04 | 0.35±0.03 | 0.07±0.04 | 1.43±0.05 | 0.23±0.02 |
|  | NT Treated | 99.00±0.17 | 0.35±0.18 | 0.09±0.05 | 0.38±0.05 | 0.20±0.02 |
|  | A7 Treated | 54.33±9.50 | 34.07±7.12 | 9.61±3.08 | 1.26±0.31 | 0.72±0.18 |

# Supplementary Table 3. Editing efficiencies with A-to-G conversion at off-target loci in the neuroretina of rd10 mice (%)

|  | OT1 | OT2 | OT3 | OT4 | OT5 | OT6 | OT7 | OT8 |
| --- | --- | --- | --- | --- | --- | --- | --- | --- |
| BSS | 0.39±0.05 | 1.38±0.25 | 0.62±0.12 | 0.83±0.18 | 1.39±0.20 | 0.60±0.05 | 1.44±0.08 | 1.34±0.17 |
| NT Treated | 0.32±0.02 | 1.36±0.28 | 0.59±0.04 | 0.71±0.16 | 1.30±0.09 | 0.60±0.04 | 1.99±0.19 | 1.32±0.09 |
| A7 Treated | 0.33±0.03 | 1.41±0.20 | 0.61±0.10 | 1.52±0.12 | 1.52±0.15 | 0.65±0.07 | 1.87±0.16 | 1.45±0.12 |

# Supplementary Table 4. The main primers used for deep sequence

| Primers | sequence |
| --- | --- |
| Pde6b-RD10-CHECKF | AGTAGGGTAAACATGGTCTG |
| Pde6b-RD10-CHECKR | GCTTGGATAGGCTCTGATAT |
| OT1-CHECKF | TCCTGCTGATTGTCATGTC |
| OT1-CHECKR | TGAAGTAGGCACCAGAGAT |
| OT2-CHECKF | GGAAATCTGGGTTAAGAACTC |
| OT2-CHECKR | CGACAACAAACAAAGTAACC |
| OT3-CHECKF | AGAGGAAGAAGATCCAACTT |
| OT3-CHECKR | CAGGAAGGAGCATATTATGT |
| OT4-CHECKF | CTAATGGCAGGTAACAGAGA |
| OT4-CHECKR | AGGAGTCAAGAACACAAGAA |
| OT5-CHECKF | CCTAAGATATTGTTCTGAGACC |
| OT5-CHECKR | CTTCCTGAGCAAGCATCC |
| OT6-CHECKF | CGGTATCTGGCAGGTATTC |
| OT6-CHECKR | AGGACTTCACTGGCATGA |
| OT7-CHECKF | CTGTGGCAGAGGATGGAT |
| OT7-CHECKR | TCCTTGTGAGCTTGTATGC |
| OT8-CHECKF | AGGCACCAAGAGAACAAG |
| OT8-CHECKR | ATTACAGCAAGCAAGAGGA |

# Supplementary Table 5. The on-target and off-target sequence of Pde6b in *rd10* mouse

|  | sequence |
| --- | --- |
| On-Target | GTGGCACCAGTTGTGATAGG |
| OT1 | AaCtGTGGCACCAGgTGTGA |
| OT2 | AGCCtTGGCcCCAGTTGaGA |
| OT3 | AGCCcTGGCtCCAGTTGTtA |
| OT4 | AGCCcTGGCtCCAGTTGTtA |
| OT5 | AGCCGTGGgACCAGgTGaGA |
| OT6 | AGCCGTGGtcCCAGTgGTGA |
| OT7 | AGaCcTGcCACCAGTTGTGA |
| OT8 | AGatGTGGCACCAGTTGgGA |

Fig. S1 Preservation of cones in rd10 mice after ABE treatment at P35. **a** Immunofluorescence analysis of representative eye cross-sections. Blue indicates DAPI and red indicates cone arrestin. ONL, outer nuclear layer. **b** Representative photopic ERG waveforms. **c** left, Averaging photopic a-wave amplitudes. * *P* < 0.05, ** *P* < 0.01, one-way ANOVA tests with Tukey’s multiple comparisons. Overall P value of ANOVA is 0.0079 and P value is 0.0387 for A7 treated vs. NT treated mice. right, Averaging photopic b-wave amplitudes. *** *P* < 0.001, one-way ANOVA tests with Tukey’s multiple comparisons. Overall P value of ANOVA is < 0.0001. P value is < 0.0001 for A7 treated vs. NT treated mice. The numbers of eyes were as follows: WT, n = 10; BSS treated, n = 10; NT treated, n = 10; and A7 treated, n = 12. Means ± s.d. are shown. Source data are provided as a Source Data file.

Fig. S2 Rescue of retinal structure and visual function in rd10 mice after ABE treatment at P90. **a** Representative eye section of an AAV-ABE-A7-treated *rd10* mouse at P90 with H&E staining. GCL, ganglion cell layer; INL, inner nuclear layer; ONL, outer nuclear layer. **b** Representative scotopic ERG traces in WT, BSS treated, NT treated, and A7 treated mice at P90. **c** Quantification of scotopic a-wave amplitudes from each group at P35. * *P* < 0.05 ,** *P* < 0.01, two-way ANOVA tests with Tukey’s multiple comparisons. Asterisks indicate significant differences between A7 treated and NT treated mice. P values are 0.0407 at -2, 0.0251 at -1.5, 0.0590 at -1.0, 0.0435 at -0.5, 0.0019 at 0, 0.0017 at 0.5, and 0.0047 at 1.0 light intensities. **d** Quantification of scotopic b-wave amplitudes from each group at P35. * *P* < 0.05, ** *P* < 0.01, *** *P* < 0.001, two-way ANOVA tests with Tukey’s multiple comparisons. Asterisks indicate significant differences between A7 treated and NT treated mice. P values are 0.0098 at -2, 0.0028 at -1.5, 0.01 at -1.0, 0.0009 at -0.5, and < 0.0001 at higher light intensities. The numbers of eyes were as follows: WT, n = 6; BSS treated, n = 6; NT treated, n = 6; and A7 treated, n = 10. Means ± s.d. are shown. Source data are provided as a Source Data file.

Fig. S3 Improvement of vision-guided behavior of rd10 mice after dual-AAV SpRY-ABE8e treatment at P90. **a** Representative swimming routes on day 4 from each group at P90. **b** Quantification of the success rate to locate the platform within 1 min from day 1 to day 4. *** *P* < 0.001, two-way ANOVA tests with Tukey’s multiple comparisons. Asterisks indicate significant difference between A7 treated and NT treated mice at day 4. P value is < 0.0001. **c** Quantification of the escape latency from day 1 to day 4. *** *P* < 0.001, two-way ANOVA tests with Tukey’s multiple comparisons. Asterisks indicate significant difference between A7 treated and NT treated mice at day 4. P value is 0.0006. **d** Quantification of the total path length from day 1 to day 4. ** *P* < 0.01, two-way ANOVA tests with Tukey’s multiple comparisons. Asterisks indicate significant difference between A7 treated and NT treated mice at day 4. P value is 0.0034. The numbers of mice were as follows: WT, n = 3; BSS treated, n = 3; NT treated, n = 3; and A7 treated, n = 3. Each mouse completed 8 trials on day 1 to day 3 and 4 trials on day 4. Data are shown as the means ± sem. Source data are provided as a Source Data file.

Fig. S4 Quantification of scotopic b-wave amplitudes of A7-treated rd10 mice at different time points. 5-week group, n=12 eyes; 3-month group, n = 10 eyes. Means ± s.d. are shown. Source data are provided as a Source Data file.

Fig. S5 Histology of representative rd10 mouse retina from P1W to P4W. **a** Top, 12/12-hour cyclic light rearing; Bottom, 24h dark rearing. Scale bars, 50 μm. **b** Quantification of rows of outer nuclear layer (n = 4 eyes for each group). *** *P* < 0.001, two-way ANOVA tests with Tukey’s multiple comparisons. Asterisks indicate significant difference between 12/12h light/dark and 24h dark rearing *rd10* mice at postnatal week 4. P value is <0.001. GCL, ganglion cell layer, INL, inner nuclear layer, ONL, outer nuclear layer. Source data are provided as a Source Data file.
