# Supplementary material for: AAV-mediated base-editing therapy ameliorates the disease phenotypes in a mouse model of retinitis pigmentosa
Source: Nat Commun. 2023 Aug 15;14:4923. doi: 10.1038/s41467-023-40655-6 (PMC10427680; doi:10.1038/s41467-023-40655-6)
Supplement: Supplementary file 3 — Reporting Summary [file 41467_2023_40655_MOESM3_ESM.pdf]

nature portfolio

Corresponding author(s): Changhao Bi

Last updated by author(s): Jul 3, 2023

Reporting Summary

Nature Portfolio wishes to improve the reproducibility of the work that we publish. This form provides structure for consistency and transparency in reporting. For further information on Nature Portfolio policies, see our Editorial Policies and the Editorial Policy Checklist.

Statistics

For all statistical analyses, confirm that the following items are present in the figure legend, table legend, main text, or Methods section.

n/a	Confirmed	
☐	☒	The exact sample size (*n*) for each experimental group/condition, given as a discrete number and unit of measurement
☐	☒	A statement on whether measurements were taken from distinct samples or whether the same sample was measured repeatedly
☐	☒	The statistical test(s) used AND whether they are one- or two-sided<br>*Only common tests should be described solely by name; describe more complex techniques in the Methods section.*
☒	☐	A description of all covariates tested
☐	☒	A description of any assumptions or corrections, such as tests of normality and adjustment for multiple comparisons
☐	☒	A full description of the statistical parameters including central tendency (e.g. means) or other basic estimates (e.g. regression coefficient) AND variation (e.g. standard deviation) or associated estimates of uncertainty (e.g. confidence intervals)
☐	☒	For null hypothesis testing, the test statistic (e.g. *F*, *t*, *r*) with confidence intervals, effect sizes, degrees of freedom and *P* value noted<br>*Give P values as exact values whenever suitable.*
☒	☐	For Bayesian analysis, information on the choice of priors and Markov chain Monte Carlo settings
☒	☐	For hierarchical and complex designs, identification of the appropriate level for tests and full reporting of outcomes
☒	☐	Estimates of effect sizes (e.g. Cohen's *d*, Pearson's *r*), indicating how they were calculated

Our web collection on statistics for biologists contains articles on many of the points above.

Software and code

Policy information about availability of computer code

Data collection	Illumina Hiseq Control Software (2.7), Amersham Imager 600, Olympus BX53, Leica SP8 Lightning System, RETIanimal System, EthoVision XT Tracking System.
Data analysis	BWA (version 0.7.12), Excel (Professional Plus 2013), Prism (9.5.0).

For manuscripts utilizing custom algorithms or software that are central to the research but not yet described in published literature, software must be made available to editors and reviewers. We strongly encourage code deposition in a community repository (e.g. GitHub). See the Nature Portfolio guidelines for submitting code & software for further information.

Data

Policy information about availability of data

All manuscripts must include a data availability statement. This statement should provide the following information, where applicable:

- Accession codes, unique identifiers, or web links for publicly available datasets
- A description of any restrictions on data availability
- For clinical datasets or third party data, please ensure that the statement adheres to our policy

There is no restriction on data associated with this study. High-throughput sequencing data have been deposited in the NCBI database (accession code PRJNA884754). Source data are provided with this paper.

Research involving human participants, their data, or biological material

Policy information about studies with human participants or human data. See also policy information about sex, gender (identity/presentation), and sexual orientation and race, ethnicity and racism.

Reporting on sex and gender	N/A
Reporting on race, ethnicity, or other socially relevant groupings	N/A
Population characteristics	N/A
Recruitment	N/A
Ethics oversight	N/A

Note that full information on the approval of the study protocol must also be provided in the manuscript.

Field-specific reporting

Please select the one below that is the best fit for your research. If you are not sure, read the appropriate sections before making your selection.

☒ Life sciences ☐ Behavioural & social sciences ☐ Ecological, evolutionary & environmental sciences

For a reference copy of the document with all sections, see nature.com/documents/nr-reporting-summary-flat.pdf

Life sciences study design

All studies must disclose on these points even when the disclosure is negative.

Sample size	No statistical methods were used to predetermine sample size for experiments. Sample sizes for mice experiments were based on prior experience of suitable n numbers and a minimum of n of 3 animals for each condition (Suh et al. 2021, doi:10.1038/s41551-020-00632-6). The sample size for the in vitro cell study was at least 3 replicates per group based on prior experience (Zhao et al. 2021, doi: 10.1038/ s41587-020-0592-2;).These sample sizes have been chosen in accordance with the standards of the field, which have been sufficient to draw conclusions and to achieve statistical significance.
Data exclusions	The mice with substantial complications from the injection procedure, such as hemorrhages or damage to the lens, were excluded from the study and subsequent analysis.
Replication	The in vitro experiments were repeated independently at least three times, with similar results. All experiments involving animals were performed in at least three independent mice and similar results were observed.
Randomization	The mice were allocated to either treated or controal group randomly.
Blinding	The researchers were not blinded to group allocation as the subsequent experiments were carried by the same researchers from the beginning.

Reporting for specific materials, systems and methods

We require information from authors about some types of materials, experimental systems and methods used in many studies. Here, indicate whether each material, system or method listed is relevant to your study. If you are not sure if a list item applies to your research, read the appropriate section before selecting a response.

Materials & experimental systems

n/a	Involved in the study	
☐	☒	Antibodies
☐	☒	Eukaryotic cell lines
☒	☐	Palaeontology and archaeology
☐	☒	Animals and other organisms
☒	☐	Clinical data
☒	☐	Dual use research of concern
☒	☐	Plants

Methods

n/a	Involved in the study	
☒	☐	ChIP-seq
☒	☐	Flow cytometry
☒	☐	MRI-based neuroimaging

Antibodies

Antibodies used	rabbit anti-PDE6β antibody (catalog PA1-722; Thermo Fisher), rat anti-HA antibody (catalog 11867431001, Roche) and rabbit anti-β-actin antibody (catalog 3779, ProSci), rabbit anti-rhodopsin antibody (catalog 14825S, CST), rabbit anti-cone arrestin antibody (catalog AB15282, Millipore), goat anti-rat IgG (H+L) antibody (catalog SA00001-15, Proteintech), goat anti-rabbit IgG (H+L) antibody (catalog SA00001-2, Proteintech), goat anti-rat IgG (H+L) antibody (catalog A-11006, Thermo Fisher) and goat anti-rabbit IgG (H+L) antibody (catalog A-11012, Thermo Fisher).
Validation	Each antibody used in this study has been validated by its manufacturers. The information was posted on the product websites.

Eukaryotic cell lines

Policy information about cell lines and Sex and Gender in Research

Cell line source(s)	HEK293T cells (from ATCC)
Authentication	The cell lines were not authenticated.
Mycoplasma contamination	The cell lines tested negative for mycoplasma contamination.
Commonly misidentified lines (See ICLAC register)	The study did not involve commonly misidentified lines.

Animals and other research organisms

Policy information about studies involving animals; ARRIVE guidelines recommended for reporting animal research, and Sex and Gender in Research

Laboratory animals	Pigmented rd10 mice and C57BL/6J mice were housed at the animal facility of Shanghai General Hospital in 12/12-h light/dark cycles. The temperature ranged from 23-25°C, and the humidity from 40% to 60%. The mice were fed with normal chow diet.
Wild animals	The study did not involve wild animals.
Reporting on sex	Sex was not considered in study design.
Field-collected samples	The study did not involve samples collected from the field.
Ethics oversight	All animal procedures were approved by the Institutional Review Board of Shanghai Jiao Tong University and were conducted in accordance with the Association for Research in Vision and Ophthalmology Statement for the Use of Animals in Ophthalmic and Visual Research.

Note that full information on the approval of the study protocol must also be provided in the manuscript.
